# Draft Genome Sequence of *Desulfotignum phosphitoxidans* DSM 13687 Strain FiPS-3

**DOI:** 10.1128/genomeA.00227-13

**Published:** 2013-05-23

**Authors:** Anja Poehlein, Rolf Daniel, Diliana D. Simeonova

**Affiliations:** Genomic and Applied Microbiology and Goettingen Genomics Laboratory, Georg-August University Goettingen, Göttingen, Germanya; Laboratory of Microbial Ecology, University of Konstanz, Konstanz, Germanyb

## Abstract

We report the 5.008-Mbp assembled draft genome sequence of *Desulfotignum phosphitoxidans* strain FiPS-3 (DSM 13687), which gains metabolic energy from the oxidation of phosphite to phosphate. Its genome provides insights into the composition and architecture of the phosphite-utilizing and energy-transducing systems required to live with phosphite as electron donor.

## GENOME ANNOUNCEMENT

*Desulfotignum phosphitoxidans* DSM 13687 strain FiPS-3 is an autotrophic anaerobic bacterium that is able to utilize phosphite as an electron donor to gain metabolic energy for growth and CO_2_ as the only carbon source (1).

The requirements of living cells for phosphorus are usually covered by phosphate and phosphate esters, but alternatively, some bacterial species can assimilate reduced phosphorus compounds under phosphate starvation (2, 3). Reduced inorganic P-compounds such as hypophosphite (+I), phosphite (+III), or organophosphonates can be transported into the cells, oxidized to phosphate (+V), and incorporated into the cell biomass (4–7). The phosphite oxidation pathways for assimilation purposes have been well documented, but very little is known about the energetic side of the process.

The genome sequencing and the reconstruction of *D. phosphitoxidans* DSM 13687 metabolism are opening a new field for exploration of a microbial lifestyle, which delineates it from all others known so far.

For sequencing, chromosomal DNA of *D. phosphitoxidans* strain FiPS-3 was isolated with the MasterPure complete DNA purification kit (Epicenter, Madison, WI). Extracted DNA was used in a combined approach using the 454 GS-FLX TitaniumXL system (titanium GS70 chemistry, Roche Life Science, Mannheim, Germany) and the Genome Analyzer II (Illumina, San Diego, CA). Shotgun libraries were prepared according to the manufacturer's protocols, resulting in 176,236 reads for 454 shotgun sequencing (13.76 × coverage) and 5,124,938 112-bp paired-end Illumina reads (102.45 × coverage). The initial hybrid *de novo* assembly performed employing the MIRA software resulted in 149 contigs. To close the gaps we used PCR-based techniques and Sanger sequencing (8), BigDye 3.0 chemistry, and an ABI3730XL capillary sequencer (Applied Biosystems, Life Technologies GmbH, Darmstadt, Germany). Gap closure was done using the Gap4 (v.4.11) software (9). The genome of *D. phosphitoxidans* strain FiPS-3 consists of a circular chromosome and a circular plasmid with sizes of 4.999 Mb and 7.7 kb, respectively, and an overall G+C content of 51.26 mol%. Yacob and Glimmer (10) software tools were used for automatic gene prediction, while RNAmmer and tRNAscan were used for the identification of rRNA and tRNA genes, respectively (11, 12). The functional annotation of the protein-coding genes was initially carried out with the IMG/ER (Intergrated Microbial Genomes/Expert Review) system (13) and manually curated by using the Swiss-Prot, TREMBL and InterPro databases (14). As a result, we obtained a total of 4,699 genes, 4,646 of which are protein coding, and 76.69% of the open reading frames (ORF) were assigned to functions. Two complete rRNA clusters and 47 tRNA genes, including those for selenocysteine incorporation, were identified. Autotrophic CO_2_ assimilation proceeds through the Wood-Ljungdahl pathway. In addition to the previously described *ptx-ptd* operon involved in phosphite uptake and oxidation (7), the genome contains a C-P lyase operon for organophosphonate uptake and utilization, with an unusual structure in comparison to other described C-P lyase operons.

### Nucleotide sequence accession numbers.

This whole-genome shotgun project has been deposited at DDBJ/EMBL/GenBank under the accession number APJX00000000. The version described in this paper is the first version, APJX01000000.
